# Michelin Tire Baby Syndrome: A Rare Case with Review of Literature

**DOI:** 10.7759/cureus.5619

**Published:** 2019-09-10

**Authors:** Farheen Malik, Laraib Malik, Sina Aziz, Jawad Ahmed, Faryal Tahir

**Affiliations:** 1 Internal Medicine, Dow University of Health Sciences, Karachi, PAK; 2 Pediatrics, Abbasi Shaheed Hospital, Karachi, PAK; 3 Pediatrics, Karachi Medical and Dental College, Karachi, PAK

**Keywords:** michelin tire baby syndrome, skin folds, congenital disorders, mtbs

## Abstract

Michelin tire baby syndrome (MTBS) is a benign hamartomatous condition with ring-like lesions present on the limbs and trunk. MTBS is a rare genodermatosis. According to our search, only 20 cases have been reported. We present a case of a six-month-old female child, with complaints of fever and seizures. Since birth, she had asymptomatic multiple, asymmetric skin folds on all four limbs, resembling “Michelin Man” logo of the French tire manufacturer. She had microcephaly with characteristic round face hypertelorism, depressed nasal bridge, hypertrichosis with low set ears, a thin down-turned vermillion border of the upper lip, and a short neck. MRI was normal. Clinically, the diagnosis of MTBS was made. In addition, the parents were counseled about the self-limiting course of this disorder. MTBS itself might not be a single disorder but may manifest as a clinical finding associated with other disorders; therefore, a regular follow up of these patients is usually advised.

## Introduction

Circumferential skin fold is an infrequent finding at birth. In Michelin tire baby syndrome (MTBS), a benign hamartomatous condition, numerous, symmetric, ring-like lesions are present on the limbs and trunk. MTBS is a rare genodermatosis (Mendelian Inheritance in Man (MIM): 156610). According to our knowledge approximately 20 cases have been reported to date [1]. It is characterized by generalized folding of additional skin that may be solitary [2] or may be associated with supplementary phenotypic abnormalities [3]. Therefore, it is imperative for clinicians to carefully exclude syndromes and identify phenotypic abnormalities. Here we present a case of a six-month-old female child with MTBS.

## Case presentation

A six-month-old female child, vaccinated up to her age, presented in the ED of Abbasi Shaheed Hospital, Karachi with complaints of fever, chest congestion, vomiting, and seizures. High-grade fever developed gradually and was continuous. She had labored breathing due to chest congestion and also had a history of nonprojectile vomiting, in small amounts, associated with intake of food. The seizures were unilateral, involving the right side of her body along with twitching of mouth and up-rolling of eyes, lasting for one to two minutes.

Our patient was the first product of consanguineous marriage. Her mother was a primigravida with nonsignificant prenatal history. Ultrasound scans were normal during regular antenatal visits. The patient was born full-term via normal vaginal delivery at the hospital. She had no postnatal complications. She was exclusively breastfed for up to six months of age after which weaning was started. Her developmental milestones were delayed; she achieved social smile at fifth week and neck holding at sixth month of life. She had no family history of fits or any other chronic illness.

**Figure 1:**
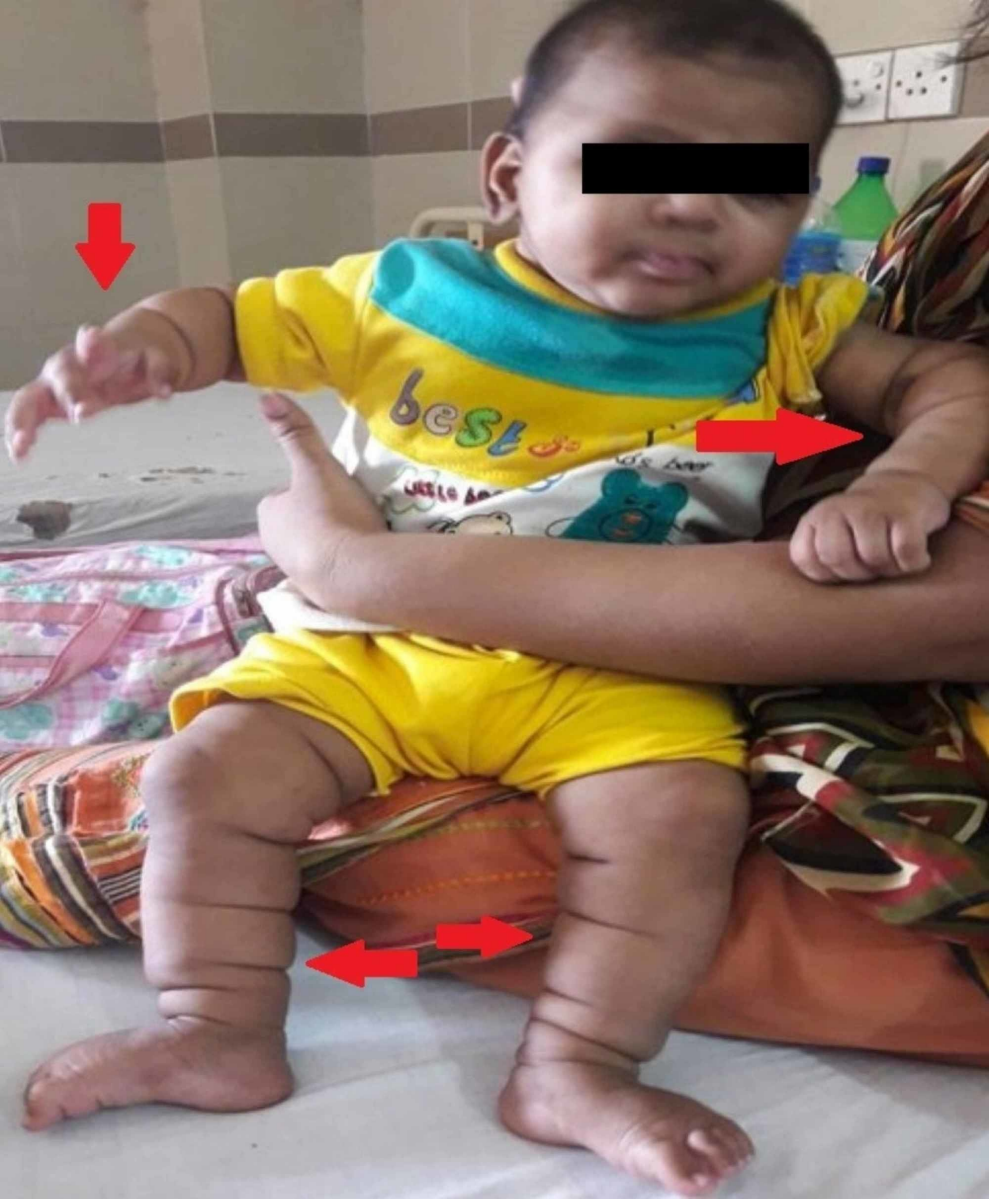
Multiple deep gyrate symmetrical skin folds present on the upper and lower limbs.

**Figure 2:**
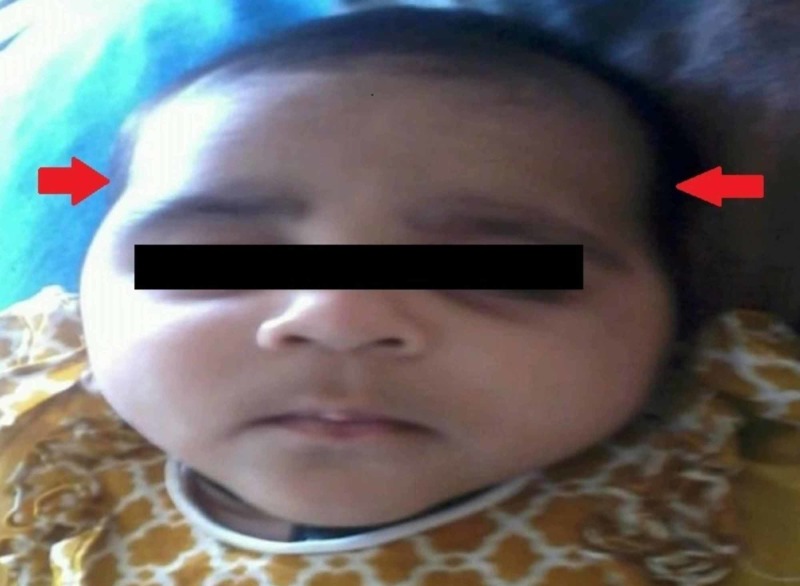
Microcephaly with small anterior fontanelle.

**Figure 3:**
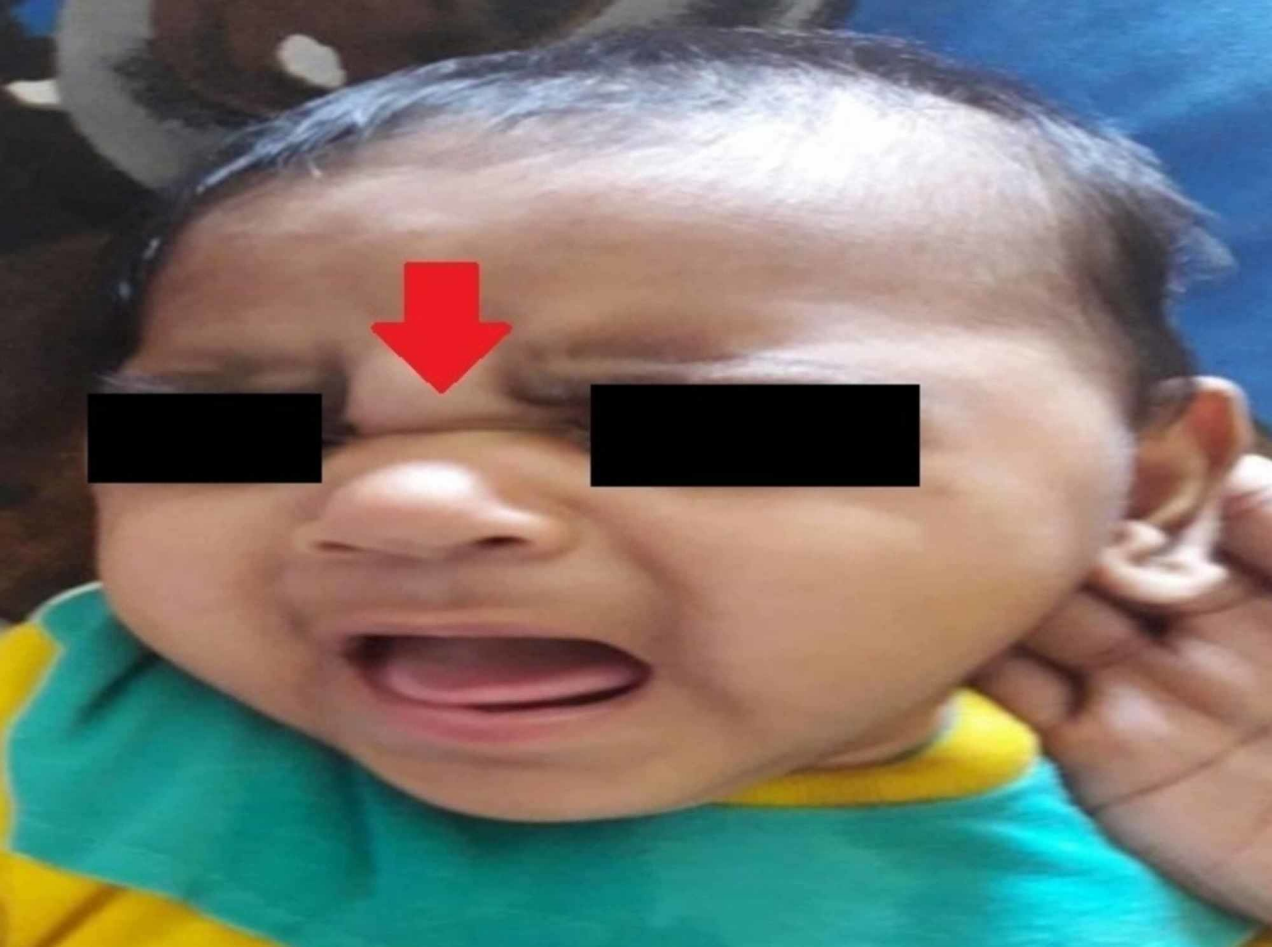
Depressed nasal bridge.

**Figure 4:**
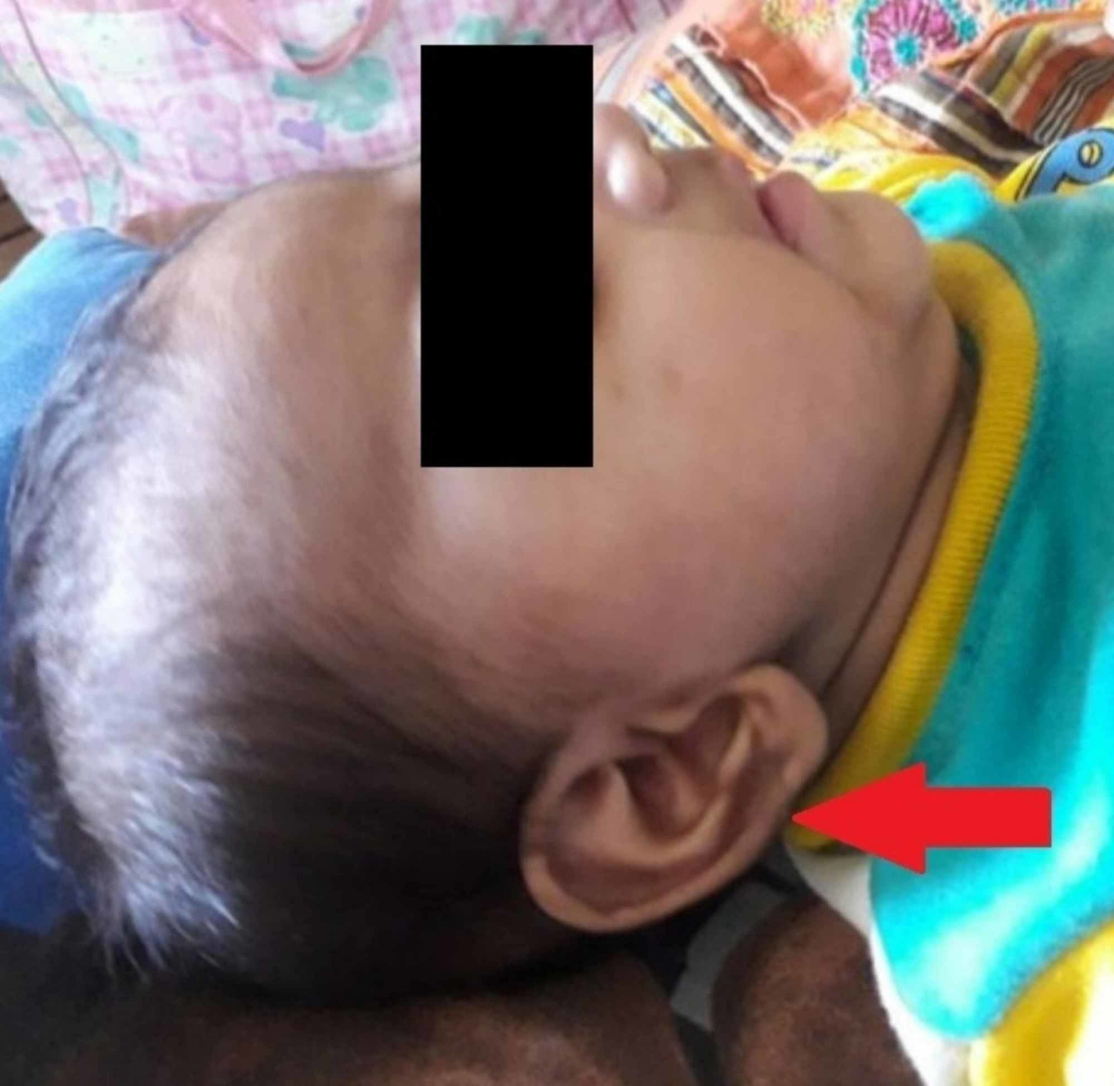
Hypertrichosis with low set ears.

General physical examination revealed a healthy baby with multiple, symmetric, deep, gyrate skin folds on the upper and lower limbs (Figure 1) resembling “Michelin Man” logo of the French tire manufacturer. The folds were present since birth and were asymptomatic without causing any physical discomfort to the child. The patient had microcephaly with small anterior fontanelle (Figure 2), her fronto-occipital circumference (FOC) was 37 cm below the 5th centile. She presented with a characteristic round face with hypertelorism, depressed nasal bridge (Figure 3), hypertrichosis with low set ears (Figure 4), thin down-turned vermillion border of the upper lip, and a short neck. Anthropometric measurements were between 5th and 10th centile. Her body weight and length was 6.5 kg and 65 cm, respectively. On neurological examination, the child did not follow commands and showed no response to voice and clapping either. Rest of the examinations were unremarkable.

Considering the presenting complaints and clinical examination, differential diagnosis of MTBS, constriction ring syndrome (CRS), and Beare-Stevenson syndrome (BSS) was made.

The blood peripheral film showed anisocytosis and hypochromic RBCs. Thyroid profile was normal. MRI was also unremarkable. Skin biopsy could not be performed due to lack of resources. Clinically, the diagnosis of MTBS was made. The patient was treated successfully with dexamethasone, vancomycin, ceftriaxone, and diazepam. Meningitis protocol was followed as lumbar puncture (LP) was refused. In addition, the parents were also counseled about the self-limiting course of this disorder. She was discharged after 10 days of treatment but was advised to follow up.

## Discussion

The term Michelin tire baby syndrome was first coined by Ross [2] for an infant having ring-like symmetrical folds of skin on the limbs. This was named because children with this disease resembled the mascot of Michelin Tire Company based in France. Bass et al. found that this disorder was familial and had an autosomal mode of inheritance. The skin folds were present in seven individuals of four generations with three cases of male to male inheritance [4]. The pathogenesis is yet not clear, however, the chromosomal anomalies found in this condition are deletion of the short arm of chromosome 11 [3] and a paracentric inversion of the long arm of chromosome 7 [5]. In our case, the patient was a female, with no family history of this disorder. Unfortunately, due to lack of funding, karyotyping and any other chromosomal analysis could not be performed.

The prominent features of MTBS include multiple, asymptomatic, circumferential folds resembling skin bands. The most common site of involvement are extremities; other sites include the trunk, palms, and sometimes soles. Bilateral epicanthic folds, low set ears, cleft palate, and lips with hypoplastic teeth and mandible may also be present [6]. In our case, multiple symmetric deep gyrate skin folds were present on the upper and lower limbs. These folds were asymptomatic and did not cause any physical discomfort to the child. Other associated features were low set ears, high arched palate, and micrognathia. She also had a down turned vermillion border of the upper lip.

In the literature, other reported anomalies include left-sided hemihypertrophy, hemiplegia and microcephaly, and smooth muscle hamartoma which can be diffuse. Hypertrichosis is usually observed. MTBS is associated with multiple congenital anomalies including craniofacial anomalies, hypoplastic scrotum, and inguinal or an umbilical hernia [7]. Gardner associated MTBS with psychomotor retardation, epilepsy, and joint hypermobility [3]. A male child with a case of undescended testes and associated abnormal testicular histology has also been reported [8]. Keeping this in view, our patient had hypertrichosis, broad and depressed nasal bridge, hypertelorism, and microcephaly. She also showed delayed development of milestones.

Dermatomegaly may be caused due to diffuse lipomatous nerve involving deeper dermis. Hence the name 'congenital diffuse lipomatosis' has been given to this condition [2]. Scarring, instead of increased adipocytes or muscle fibers, has been reported where an underlying smooth muscle hamartoma can also be present [6]. According to Sato et al., one of the pathogenetic factors may be abnormal elastic fiber formation leading to fragmentation of elastic fibers along with increased smooth muscle mass [9]. But in our case, skin biopsy could not be done due to some financial constraints.

The MTBS itself might not be a single disorder but may manifest as a clinical finding associated with other disorders, for example, BSS shows dermatomegaly limited to forehead, scalp, face, and neck [10]. Circumferential skin folds have been made a component of multiple congenital anomalies/mental retardation (MCA/MR) syndrome and hearing impairment, undescended testis, circumferential skin creases, and mental handicap (HITCH) syndrome [11]. It appears that the affected individuals are normal and the skin folds are self-limiting, however, in familial cases, remnants of deep skin folds may be present in older individuals.

The skin creases on a patient of MTBS appear similar to congenital skin creases which are not associated with any pathology. Skin biopsy is warranted to identify a variety of underlying changes that may be present in MTBS patient [2, 6]. Circumferential skin folds in MTBS are, however, associated with mental retardation and a number of developmental and facial anomalies previously mentioned. These anomalies have been suggested as part of the diagnostic criteria of MTBS [12]. In the nonpathological cases of congenital skin creases, no such associations are found in the individuals and the skin biopsy is usually normal.

Circumferential skin folds are a rare finding in the newborns, especially if the folds are one or few constrictions involving only the limbs. Further, they may be a part of CRS [13] in which all the remaining features of MTBS are absent, including genetic testing.

Skin folds may be the clinical finding of a disorder like BSS, associated with the dermatology localized to scalp, forehead, face and neck, hearing impairment, undescended testes, and circumferential skin creases. There is a premature fusion of skull bones (craniosynostosis), which prevents normal growth of the skull and in turn affects shape of the head and face. As a result, a cloverleaf-shaped skull, wide-set and bulging eyes, ear abnormalities, and an underdeveloped jaw are frequently present. Also, brain growth is affected causing delayed development and intellectual disability. It may also be associated with genital and anal abnormalities [10, 14].

## Conclusions

In conclusion, MTBS may manifest with or without any congenital abnormalities. It may also be a part of a syndrome and the patient may manifest delayed mental development, therefore, a regular follow up for a patient presenting with MTBS is advised.
